# Investigating Influenza Virus Polymerase Activity in Feline Cells Based on the Influenza Virus Minigenome Replication System Driven by the Feline RNA Polymerase I Promoter

**DOI:** 10.3389/fimmu.2022.827681

**Published:** 2022-05-26

**Authors:** Gang Lu, Feiyan Zheng, Jiajun Ou, Xin Yin, Shoujun Li

**Affiliations:** ^1^College of Veterinary Medicine, South China Agricultural University, Guangzhou, China; ^2^State Key Laboratory of Veterinary Biotechnology, Harbin Veterinary Research Institute, The Chinese Academy of Agricultural Sciences, Harbin, China; ^3^Guangdong Provincial Key Laboratory of Prevention and Control for Severe Clinical Animal Diseases, Guangzhou, China; ^4^Guangdong Technological Engineering Research Center for Pet, Guangzhou, China

**Keywords:** RNA polymerase I promoter, feline, influenza virus, polymerase activity, minigenome replication system

## Abstract

Emerging influenza virus poses a health threat to humans and animals. Domestic cats have recently been identified as a potential source of zoonotic influenza virus. The influenza virus minigenome replication system based on the ribonucleic acid (RNA) polymerase I (PolI) promoter is the most widely used tool for investigating polymerase activity. It could help determine host factors or viral proteins influencing influenza virus polymerase activity *in vitro*. However, influenza virus polymerase activity has never been studied in feline cells thus far. In the present study, the feline RNA PolI promoter was identified in the intergenic spacer regions between adjacent upstream 28S and downstream 18S rRNA genes in the cat (*Felis catus*) genome using bioinformatics strategies. The transcription initiation site of the feline RNA PolI promoter was predicted. The feline RNA PolI promoter was cloned from CRFK cells, and a promoter size of 250 bp contained a sequence with sufficient PolI promoter activity by a dual-luciferase reporter assay. The influenza virus minigenome replication system based on the feline RNA PolI promoter was then established. Using this system, the feline RNA PolI promoter was determined to have significantly higher transcriptional activity than the human and chicken RNA PolI promoters in feline cells, and equine (H3N8) influenza virus presented higher polymerase activity than human (H1N1) and canine (H3N2) influenza viruses. In addition, feline myxovirus resistance protein 1 (Mx1) and baloxavir were observed to inhibit influenza virus polymerase activity *in vitro* in a dose-dependent manner. Our study will help further investigations on the molecular mechanism of host adaptation and cross-species transmission of influenza virus in cats.

## Introduction

Influenza A virus, classified in the *Orthomyxoviridae* family, is an enveloped virus with a negative sense ssRNA genome. Its genome consists of eight segments encoding at least 14 viral proteins. Within the virion, each of the viral RNA (vRNAs) is associated with polymerase subunits (PB1, PB2, and PA) and NP to form viral ribonucleoprotein (vRNP) complexes. After the influenza virus enters the host cell, the vRNP is released into the cytoplasm and then transported into the nucleus. In the nucleus, the RNP is responsible for capped RNA-primed mRNA synthesis and unprimed replication of vRNAs following these steps: (−)vRNA → (+)cRNA → (−)vRNA. After the influenza virus enters the host cell, a major host range restriction is determined by the interaction between viral ribonucleoprotein and cellular cofactors in the nucleus, which can be reflected as influenza virus polymerase activity.

To study influenza virus polymerase activity *in vitro*, the influenza virus minigenome replication system has been developed under the control of the RNA polymerase I (PolI) promoter from several major hosts (human, dog, chicken, horse, and pig) of influenza virus (1–7). However, the RNA PolI promoter is species specific and cannot be recognized efficiently by the RNA PolI enzyme in distantly related species (8, 9). It has been reported that the influenza virus minigenome replication system based on the bacteriophage T7 promoter could work in a variety of cell lines, including human-, dog-, and quail-derived cell lines (10). However, the influenza virus minigenome replication system based on the T7 promoter depends on the expression of the T7 RNA polymerase protein in the transfected cells. Accordingly, an additional T7 RNA polymerase expression plasmid should be included in the influenza virus minigenome replication system based on the T7 promoter compared with the RNA PolI promoter. Alternatively, an additional cell line constantly expressing T7 RNA polymerase should be established. This limited the application of the influenza virus minigenome replication system based on the T7 promoter. The influenza virus minigenome replication system was established based on mimicking the replication process of the genuine influenza virus. In this system, the RNA PolI enzyme recognizes the promoter and terminator sequences and synthesizes a negative-sense, virus-like vRNA. For example, in the five-plasmid influenza virus minigenome replication system developed by Hoffmann *et al.*, the antisense influenza virus-like cDNA of a reporter gene was inserted between the human RNA PolI promoter and the murine terminator sequences (1). This plasmid was transfected into 293T or Vero cells, together with eukaryotic expression plasmids expressing PB1, PB2, PA, and NP proteins. Finally, the expression level of the reporter protein was determined and used to reflect influenza virus polymerase activity. With the help of the influenza virus minigenome replication system, many host factors or amino acid positions of viral proteins influencing the cross-species transmission of influenza virus have been determined (11–15).

Domestic cats have been regarded as restraining influenza virus infection. However, since 2004, domestic cats have been reported to be infected with H3N2 canine influenza virus (CIV) (16), the 2009 pandemic H1N1 (pdm09H1N1) influenza virus (17, 18), H5N1 and H5N6 avian influenza virus (AIV) (19, 20), and H3N8 equine influenza virus (EIV) (21). Recently, the first case of human infection with H7N2 AIV *via* cat-to-human transmission was reported (22, 23). The newly emerging influenza viruses in domestic cats are of great public health concern and emphasize the potential role of cats in influenza virus circulation. However, influenza virus polymerase activity has not been studied in feline cells thus far. Our study aimed to establish an influenza virus minigenome replication system driven by the feline RNA PolI promoter and investigate influenza virus polymerase activity in feline cells.

## Materials and Methods

### Cell and Virus

Human embryonic kidney cells (293T), chicken fibroblast cells (DF-1), and Crandell Rees feline kidney cells (CRFK) were obtained from the Type Culture Collection Committee, Chinese Academy of Sciences, China. These cells were maintained in Dulbecco’s modified Eagle’s medium (DMEM, HyClone, USA) containing 10% fetal bovine serum (FBS, BI, Israel) and 100 U of penicillin and streptomycin (BI, Israel) using standard culturing conditions.

Stocks of the CIV strain A/canine/Guangdong/02/2011 (canine/GD/11, H3N2) and EIV strain A/equine/Heilongjiang/ss1/2013 (equine/HLJ/13, H3N8) were propagated in the allantoic cavities of 10-day-old specific-pathogen-free embryonated hen eggs at 37°C for 72 h. The allantoic fluid was harvested and stored at −80°C. The PB2, PB1, PA, and NP genes of canine/GD/11 and equine/HLJ/13 were cloned from allantoic fluid and used to establish the corresponding influenza virus minigenome replication system.

### Plasmid

The pHW2000 plasmid and the pHW2000 reverse genetic system for A/WSN/1933 (WSN/33, H1N1 [pHW181-PB2, pHW182-PB1, pHW183-PA, pHW184-HA, pHW185-NP, pHW186-NA, pHW187-M, and pHW188-NS]) were kindly provided by Professor Robert G. Webster, St. Jude Children’s Research Hospital. The eukaryotic expression plasmid pCAGGS contained the chicken β-actin promoter. The pCAGGs-WSN-PB2, pCAGGs-WSN-PB1, pCAGGs-WSN-PA, and pCAGGs-WSN-NP plasmids expressing PB2, PB1, PA, and NP protein of WSN/33 were constructed by amplifying the coding sequence from pHW181-PB2, pHW182-PB1, pHW183-PA, and pHW185-NP using PCR technology, respectively. The pCAGGs-GD-PB2, pCAGGs-GD-PB1, pCAGGs-GD-PA, and pCAGGs-GD-NP and pCAGGs-HLJ-PB2, pCAGGs-HLJ-PB1, pCAGGs-HLJ-PA, and pCAGGs-HLJ-NP plasmids were constructed by the same method using canine/GD/11 and equine/HLJ/13 cDNAs as PCR templates.

The human RNA PolI promoter sequence was obtained from pHW2000 by PCR. The chicken RNA PolI promoter sequence reported in a previous study was synthesized (BGI, China). The feline RNA PolI promoter sequence was amplified from the CRFK DNA template by PCR in this study (2). The artificial vRNA-luciferase reporter plasmids generating negative-strand, influenza virus-like luciferase transcripts were under the control of human, chicken, or feline RNA PolI promoters (pHUpol-LUCI, pCHpol-LUCI, and pCATpol-LUCI). The three plasmids were obtained by an *in vitro* homologous recombination approach in our previous study using the ClonExpress MultiS One-Step Cloning Kit (Vazyme, China) (6). Briefly, the CMV promoter and human RNA PolI promoter of pHW2000 were removed, and this segment was homologously recombined with one segment containing the RNA PolI promoter sequence and the other segment containing the murine terminator sequence and an antisense luciferase coding sequence flanked by the 5′ and 3′ noncoding regions (NCRs) of the NP gene of the influenza virus. The artificial vRNA-luciferase reporter plasmids generating luciferase transcripts driven by the feline RNA PolI promoters of different lengths (nt −1 to −1000, −500, −250, −125, and −60 [pCAT1000, pCAT500, pCAT250, pCAT125, and pCAT60, respectively]) were constructed using the same strategy.

The feline myxovirus resistance protein 1 (Mx1) gene was obtained from cat tissue samples by RT–PCR and then cloned into the pCAGGs plasmid (pCAGGs-FeMx1).

All recombinant plasmids were confirmed by DNA sequencing before the follow-up experiments.

### PCR

A monolayer of CRFK cells with approximately 90% confluence in a 25 cm^2^ flask was digested and harvested. The genomic DNA of CRFK cells was extracted using a Minibest Universal Genomic DNA Extraction Kit (Takara, Japan). To obtain the feline RNA PolI promoter sequence, three PCR primers targeting Contig1056.4 [AANG03041276] were designed by Oligo 7.0. The nucleotide sequence of PCR primers (5’→3’): [PCR1] GCTGTGCGCCCTCACGTC, GGTCCGGCTCAGCGTCAC; [PCR2] TCGGCGACGTCGGCAAGG, GGTCCGGCTCAGCGTCAC; and [PCR3] CAAGGGATGTGATTTGGTCGCAGTG, CCACCGCCACATCAGAACGTGTCAG. Three independent PCRs (PCR1, PCR2, and PCR3) were performed using Phanta max superfidelity DNA polymerase (Vazyme, China) and the genomic DNA of CRFK cells as a template. The following two-step PCR procedure was performed: 95°C for 3 min, 35 cycles at 95°C for 30 s, and 72°C for 90 s, followed by 1 cycle at 72°C for 5 min. The PCR fragments were purified using a universal DNA purification kit (TianGen, China) and cloned into a pCloneEZ-NRS-Blunt-Amp HC Cloning Kit (Clone smarter, USA). After transformation into *E. coli* competent cells (Weidi, China), the positive colonies were sent for Sanger sequencing from both ends (BGI, China).

### Transfection

293T, DF-1, or CRFK cell monolayers in 24-well plates were transfected with each of the pCAGG plasmids expressing PB2 (200 ng), PB1 (200 ng), PA (200 ng), and NP (300 ng), the luciferase reporter plasmid (300 ng), and an internal control thymidine kinase promoter-pRL-TK expressing *Renilla* luciferase (30 ng) (Promega, USA) to normalize transfection efficiencies using FuGENE transfection reagent (Promega, USA [ratio, wt/vol=1:3]) according to the manufacturer’s instructions. Twenty-four hours after transfection, the expression of luciferase was examined by a dual-luciferase reporter assay system (Promega, USA).

### Cell Cytotoxicity Assay

The CCK8 assay was used to determine the cytotoxicity of Baloxavir (S-033447; BXA) toward CRFK cells according to the manufacturer’s instructions (Vazyme, China). Briefly, CRFK cells in 48-well plates were left untreated or treated with baloxavir at concentrations ranging between 25×2^1^ and 25×2^-6^ μM using 2-fold change serial dilutions. The absorbance was measured using a microplate reader at 450 nm. The untreated control was set at 100%, and the survival rate (%) was calculated according to the following formula: [optical density (OD) of the treated cells - OD of blank control/OD of negative control - OD of blank control] × 100%.

### Statistical Analysis

All experiments were performed in triplicate. Statistical significance was determined using Student’s t test and calculated by GraphPad Prism software 6. A *P* value of <0.05 was considered significant (^*^, *p* < 0.05; ^**^, *p* < 0.01).

## Result

### Identification of the Transcription Initiation Site of the Feline RNA PolI Promoter

**Figure 1:**
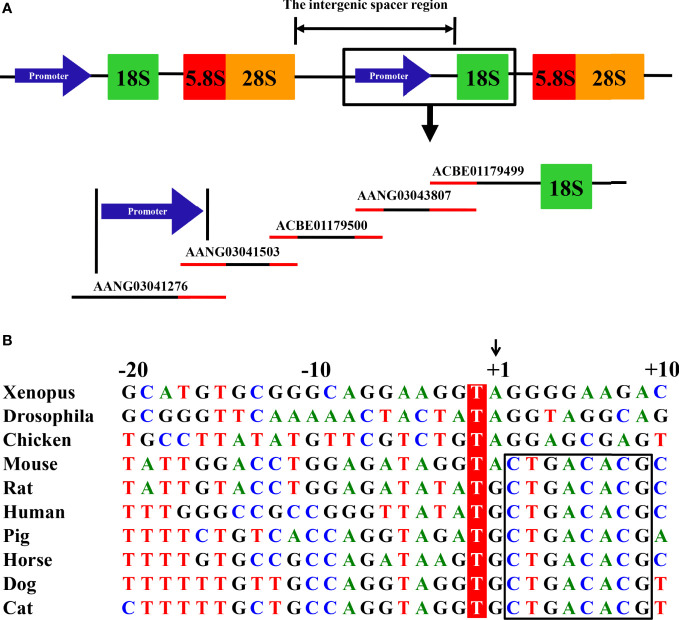
Mapping of the feline RNA PolI promoter. **(A)** Eukaryotic 18S, 5.8S, and 28S rRNA genes are organized as clusters of head-to-tail repeats. After searching the cat genome, one contig, ACBE01179499, was confirmed to contain the cat 18S rRNA gene. Through homology comparison between four other contigs located upstream of the 18S rRNA in the cat genome, two contigs, AANG03041503 and AANG03041276, were predicted to contain the cat RNA PolI promoter. The homology region between the five contigs is indicated in red. **(B)** Alignment of the nucleotide sequence from the -20 to +10 position of the predicted transcription initiation site of the feline RNA PolI promoter. The -1 position of the predicted transcription initiation site was conserved among all of the eukaryote species and was labeled in red. The predicted transcription initiation site is indicated by +1 and an arrow. The conserved +2 to +9 position among all of the mammals is indicated by a black box.

Eukaryotic 18S, 5.8S, and 28S ribosomal RNA (rRNA) genes are organized as clusters of head-to-tail repeats (**Figure 1A**). The RNA PolI promoter is located in the intergenic spacer regions between the adjacent upstream 28S and downstream 18S rRNA genes. Using the same bioinformatics method as described in determining the canine and horse RNA PolI promoter (4, 6), the human 18S rRNA nucleotide sequence (GenBank accession number: X03205) of 1869 bp in the NCBI database was first obtained. A homologous search was then performed in the database of the cat genome (*Felis catus* [domestic cat]; https://www.ncbi.nlm.nih.gov/genome/?term=cat). One genomic contig, c599102987.Contig1 (GenBank accession number: ACBE01179499) of 5150 bp, was identified containing the cat 18S rRNA sequence, with a genetic similarity of 98.5% with human 18S rRNA. Next, a homologous search was continually performed in the cat genome, and a total of four other genomic contigs with overlapping homologous regions were determined upstream of c599102987.Contig1 (Contig2853.1, [AANG03043807]; c401400926.Contig2, [ACBE01179500]; Contig1209.2, [AANG03041503]; and Contig1056.4, [AANG03041276]) (**Figure 1A**).

Although the primary sequence of the RNA PolI promoter from different species is not well conserved, the sequences around its transcription initiation sites are relatively conserved (2, 4–7). The common nucleotide sequences around the transcription initiation site of mammalian animals (-10 to +10 nt [the transcription initiation site of the RNA PolI promoter was defined as +1]) were queried in the five obtained contigs and hit the 340 to 359 nt position of Contig1209.2 and the 2411 to 2430 nt position of Contig1056.4. The base at the +2 position of the transcription initiation site of the RNA PolI promoter was different in the two contigs (Contig1209.2, T; Contig1056.4, C). The sequences around the transcription initiation site of the feline RNA PolI promoter in Contig1056.4 were provisionally used for comparison with those from other eukaryotes (**Figure 1B**). It clearly demonstrated that the transcription initiation site was A/G; the -1 position of the transcription initiation site was highly conserved and was always T; among mammals, the -18, -7, -5, -4, and +2 to+9 positions of the transcription initiation site were T, G, T, A, and CTGACACG, respectively, as indicated in previous studies (4–6).

### Cloning and Genetic Analysis of the Feline RNA PolI Promoter

After identifying the predicted transcription initiation site of the feline RNA PolI promoter, we cloned the promoter using three PCR primer pairs based on Contig1056.4, targeting the -1184 to +120, -1094 to +120, and -1081 to +38 nt positions of the predicted transcription initiation site of the feline RNA PolI promoter (**Supplementary Figure S1A**). The three PCR primer pairs were independently used in PCR to improve the possibility of obtaining the targeted DNA fragment. After agarose gel electrophoresis, three bright bands with the expected size were observed. This demonstrated that all three PCR primer pairs could amplify the targeted DNA fragments with high specificity. The sequencing result was aligned with the nucleotide sequence of Contig1056.4. A comparison of the -1200 to +100 nt position of the predicted transcription initiation site between the feline RNA PolI promoter obtained in this study and Contig1056.4 indicated 97.8% nucleotide similarity. This included seven base mutations, three base deletions, and 16 base inserts (**Supplementary Figure S1B**). Notably, the 16 base inserts were only located in two continuous regions: -831 to 854 nt and -883 to 894 nt. In addition, the +2 position of the predicted transcription initiation site of the feline RNA PolI promoter derived from CRFK cells was identical to that in Contig1056.4, further confirming the sequence alignment result in **Supplementary Figure S1B**.

### Establishment of an Influenza Virus Minigenome Replication System Driven by the Feline RNA PolI Promoter

**Figure 2:**
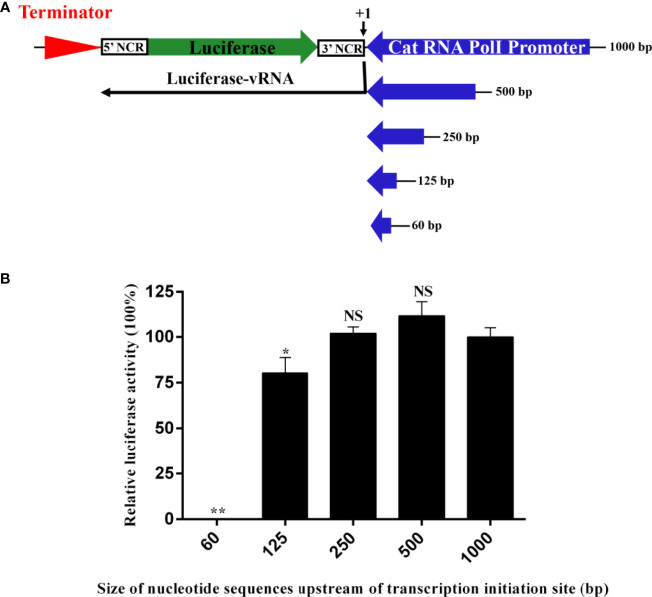
Identification of the narrowest promoter size required for the highest transcriptional activity of the feline RNA PolI promoter. **(A)** The strategy for generating artificial vRNA-luciferase reporter transcripts under the control of feline PolI promoters of different lengths. The antisense firefly luciferase coding sequence was flanked by the 5′ and 3′ noncoding sequences of the NP segment of influenza virus. Five constructs with cat PolI promoters of different lengths (60, 125, 250, 500, and 1000 bp) were established. **(B)** Identification of the narrowest promoter size required for the highest transcriptional activity of the cat RNA PolI promoter using a dual-luciferase reporter assay. pCAT60, pCAT125, pCAT250, pCAT500, and pCAT1000 (300 ng) were each individually combined with expression plasmids expressing PB1 (200 ng), PB2 (200 ng), PA (200 ng), and NP (300 ng) proteins of WSN/33 (pCAGGs-WSN-PB1, pCAGGs-WSN-PB2, pCAGGs-WSN-PA, and pCAGGs-WSN-NP, respectively). Together with pRL-TK (30 ng), these plasmids were transfected into CRFK cells in a 24-well plate using FuGENE transfection reagent (Promega, USA) according to the manufacturer’s instructions. At 24 h after transfection, dual luciferase activity was detected (Promega, USA), and the relative luciferase activity was represented as the ratio of firefly luciferase to *Renilla* luciferase. The relative luciferase activity subjected to the pCAT1000 construct was set as 100%. The results of three repeated experiments are shown. The differences in RNA PolI activity were compared with the pCAT1000 experimental group using an unpaired two-tailed Student’s *t* test (NS, not significant; ^*^p < 0.05; ^**^p < 0.01).

To construct an influenza virus minigenome replication system driven by the feline RNA PolI promoter, it is necessary to identify the size of the promoter to generate the highest transcription activity. Previous studies on the RNA PolI promoter of humans, chickens, African green monkeys, canines, horses, and pigs have indicated that this size is ≤ 500 bp (1–7). Accordingly, a luciferase reporter plasmid controlled by five feline RNA PolI promoters of 60 bp to 1000 bp was obtained (**Figure 2A**). The influenza virus minigenome replication system includes this reporter plasmid and four other pCAGG plasmids expressing the PB2, PB1, PA, and NP proteins of influenza virus. After cotransfection into CRFK cells, with the help of the feline RNA PolI promoter enzyme, a luciferase-vRNA was transcribed by the RNA PolI promoter and terminated by the downstream murine RNA PolI terminator element. The antisense luciferase-vRNA was recognized by PB2, PB1, PA, and NP proteins, and then luciferase-cRNA and luciferase-mRNA were transcribed. The results of the dual-luciferase reporter assay indicated that the feline RNA PolI promoters of 250 bp and 500 bp had the same transcriptional activity as that of 1000 bp (**Figure 2B**) (*p*>0.05). When the feline RNA PolI promoter was truncated to 125 bp, the transcriptional activity was decreased by approximately 25% (*p*<0.05). As a control, a feline RNA PolI promoter of 60 bp had no transcriptional activity (*p*<0.01). This result demonstrated that a 250 bp feline promoter contained a sequence with sufficient PolI promoter activity in CRFK cells.

### Species Specificity of the Feline RNA PolI Promoter

**Figure 3:**
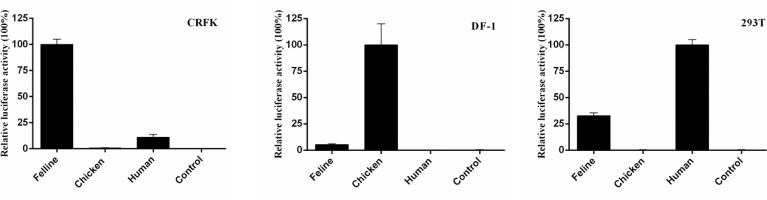
Characterization of the species specificity characteristic of the feline RNA PolI promoter. Characterization of the species specificity characteristic of the feline RNA PolI using a dual-luciferase activity assay. Briefly, pHUpol-LUCI, pCHpol-LUCI, and pCAT250 were individually combined with the plasmids pCAGGs-WSN-PB2, pCAGGs-WSN-PB1, pCAGGs-WSN-PA, pCAGGs-WSN-NP and pRL-TK and transfected into CRFK, DF-1, and 293T cells in a 24-well plate, with the same transfection and data processing method described in **Figure 2B**. As a control, an empty plasmid with no PolI promoters was included to replace pHUpol-LUCI, pCHpol-LUCI, and pCAT250 in this experiment.

Studies on humans, chickens, canines, and horses have indicated that the RNA PolI promoter is species specific (2–4, 6). To determine whether the feline RNA PolI promoter has this characteristic, three influenza virus minigenome replication systems controlled by the human, chicken, and feline RNA PolI promoters were used and tested for their transcriptional activity in 239T, DF-1, and CRFK cells (**Figure 3**). The results of the dual-luciferase reporter assay demonstrated that the three RNA PolI promoters had higher transcriptional activity in their host species-derived cells than in distantly related species-derived cells. In addition, the feline RNA PolI promoter had low transcriptional activity in DF-1 cells but had approximately 20% transcriptional activity with that of the human RNA PolI promoter in 293T cells, indicating that the feline RNA PolI promoter was active at a moderate level in 293T cells. The results demonstrated the species specificity characteristic of the feline RNA PolI promoter.

### Studying Influenza Virus Polymerase Activity in CRFK Cells

**Figure 4:**
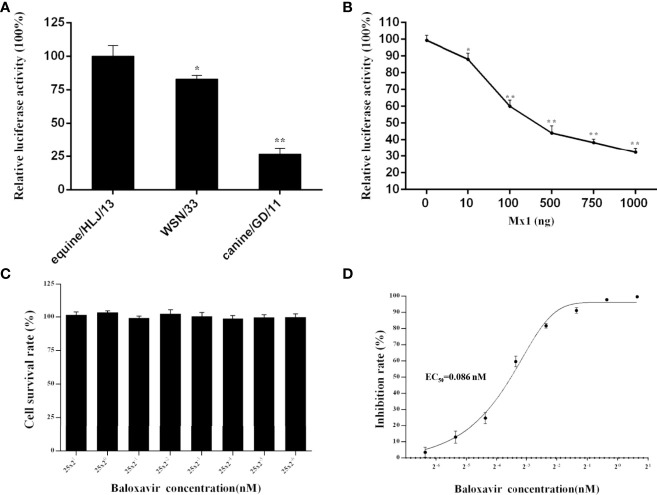
Comparison of the polymerase activity of influenza virus and the inhibitory function of feline Mx1 and baloxavir on the polymerase activity of influenza virus in CRFK cells. **(A)** The polymerase activity of three representative influenza A virus strains (H1N1 human influenza virus, WSN/1933; H3N8 EIV, equine/HLJ/13; and H3N2 CIV, canine/GD/11) in CRFK cells was estimated by dual-luciferase activity experiments. The relative luciferase activity of WSN/33 in CRFK cells was set as 100%. Each experiment was repeated three times. The differences in relative luciferase activity were compared with the WSN/33 group using an unpaired two-tailed Student’s *t* test (^*^p < 0.05; ^**^p < 0.01). **(B)** Inhibition function of feline Mx1 on the polymerase activity of influenza virus in CRFK cells. Increasing amounts (0 ng, 50 ng, 100 ng, and 200 ng) of pCAGGS-FeMx1 were cotransfected into CRFK cells together with the influenza virus minigenome replication system driven by the feline RNA PolI promoter. The relative luciferase activity subjected to 0 ng pCAGGS-FeMx1 construct was set as 100%. The differences in luciferase activity in other experimental groups were compared with the 0 ng pCAGGS-FeMx1 experimental group. Data are shown as the means ± SD from three independent experiments using an unpaired two-tailed Student’s *t* test (^*^p < 0.05; ^**^p < 0.01). **(C, D)** Inhibition function of baloxavir on the polymerase activity of influenza virus in CRFK cells. **(C)** The CCK8 assay was used to determine the cytotoxicity of baloxavir (concentrations ranging between 25×2^1^ and 25×2^-6^ μM) toward CRFK cells. The absorbance of the untreated control was set at 100%, and the treated samples were normalized to this value. Each experiment was performed in triplicate. **(D)** Twenty-four hours after baloxavir treatment, CRFK cells were transfected with the influenza virus minigenome replication system driven by the feline RNA PolI promoter. Twenty-four hours after transfection, the expression of luciferase was examined by a dual-luciferase reporter assay system. Each experiment was repeated three times. The EC_50_ was calculated by GraphPad Prism 6 software.

The newly established influenza virus minigenome replication system under the control of the feline RNA PolI promoter was applied to study the polymerase activity of three representative influenza A virus strains in CRFK cells: H1N1 human influenza virus, WSN/1933; H3N8 EIV, equine/HLJ/13; and H3N2 CIV, canine/GD/11 (**Figure 4A**). The results indicated that equine/HLJ/13 had the highest polymerase activity among the three strains; WSN/1933 and canine/GD/11 had approximately 80% (*p*<0.05) and 25% (*p*<0.01) polymerase activity of equine/HLJ/13, respectively. This demonstrated that the viral polymerase complex of equine/HLJ/13 was more adaptive to feline cells than WSN/1933 and canine/GD/11.

It has been demonstrated that human myxovirus resistance protein A (MxA) can act as an anti-influenza virus factor by specifically inhibiting polymerase activity. To investigate whether feline myxovirus resistance protein 1 (Mx1) could inhibit the polymerase activity of influenza virus, CRFK cells were transiently transfected with increasing amounts of feline Mx1 constructs together with the influenza virus minigenome replication system driven by the feline RNA PolI promoter. Feline Mx1 overexpression inhibited the polymerase activity of influenza virus in a dose-dependent manner (**Figure 4B**).

Baloxavir is a cap-dependent inhibitor of the polymerase acid protein of influenza viruses. To study the potential function of baloxavir on the polymerase activity of influenza virus in CRFK cells using the influenza virus minigenome replication system developed in the present study, the cytotoxicity of baloxavir toward CRFK cells was first estimated. The results demonstrated that baloxavir (concentrations ranging between 25×2^1^ and 25×2^-6^ μM) showed no cytotoxic effect toward CRFK cells (*p*<0.05) (**Figure 4C**). In addition, baloxavir inhibited the polymerase activity of influenza virus in a dose-dependent manner, and the EC_50_ was estimated to be 0.086 nM (**Figure 4D**).

## Discussion

The previously reported strategy for determining the transcription initiation site of the RNA PolI promoter includes the primer extension method and bioinformatics method (2–7). The latter method has been applied in the study of the African green monkey, canine, horse, and pig RNA PolI promoter (4–7). In two separate studies, the transcription initiation site of the canine RNA PolI promoter was successfully identified using both methods (3, 4). In this study, the predicted transcription initiation site of the feline RNA PolI promoter was successfully determined using a bioinformatics method. The luciferase-based feline PolI influenza reporter construct was established, and it drove transcription immediately downstream of the predicted transcription initiation site of the feline RNA PolI promoter and expressed the corresponding reporter proteins. This indicated that the predicted transcription initiation site of the feline RNA PolI promoter in this study is coincident with the actual transcription pattern.

The significantly lower activity of the human and chicken RNA PolI promoters compared with that of the feline RNA PolI promoter in CRFK cells clearly demonstrated their species specificity and indicated the necessity of using the feline RNA PolI promoter to study influenza virus polymerase activity in feline cells. Until now, the exact mechanism for the species specificity of the RNA PolI promoter has not been fully revealed. At least two trans-acting factors, the species-specific factor SL1 and the upstream binding factor (UBF), are determinants of specificity (24, 25). A phenomenon of not strict species specificity was found when investigating the feline RNA PolI promoter in 293T cells. It is possible that the feline RNA PolI promoter was more permissive to trans-acting factors in 293T cells than the chicken RNA PolI promoter.

Using the newly established influenza virus minigenome replication system, the influenza virus polymerase activity of one equine influenza virus strain (equine/HLJ/13) was higher than that of one H1N1 human influenza virus strain and one H3N2 CIV strain in CRFK cells. Previously, our experimental studies showed that cats were susceptible to equine/HLJ/13 and that the infection can be transmitted by close contact (21). All of the studied cats inoculated with equine/HLJ/13 developed overt clinical signs, virus shedding, and corresponding histopathologic changes. The high polymerase activity of equine/HLJ/13 could partly explain the molecular mechanism of its infection in cats and suggested the potential threat of EIV to cats.

As a companion animal, cats have a large population worldwide and have close contact with humans. The recently emerging feline influenza virus is a great threat to public health and indicates the necessity of investigating the factors influencing its cross-species transmission. After entering host cells, the replication ability of influenza virus can be reflected by polymerase activity. In this study, we obtained the feline RNA PolI promoter using a bioinformatics method and constructed an influenza virus minigenome replication system driven by it. The reporter system indicated that the feline RNA PolI promoter had significantly higher transcriptional activity than the human or chicken RNA PolI promoter in feline cells. The influenza virus minigenome replication system based on the feline RNA PolI promoter establishes the foundation for the further study of the host factors or viral proteins influencing the cross-species transmission of influenza virus to cats *in vitro*.

## Data Availability Statement

The datasets presented in this study can be found in online repositories. The names of the repository/repositories and accession number(s) can be found in the article/**Supplementary Materials**.

## Author Contributions

GL, XY, and SL conceived and designed the experiments; GL, FZ, JO, XY, and SL performed the experiments; GL and JO analyzed the data; GL contributed to the writing of the manuscript. All authors read and approved the final manuscript.

## Funding

This work was supported by Natural Science Foundation of Guangdong Province (2018B030311037).

## Conflict of Interest

The authors declare that the research was conducted in the absence of any commercial or financial relationships that could be construed as a potential conflict of interest.

## Publisher’s Note

All claims expressed in this article are solely those of the authors and do not necessarily represent those of their affiliated organizations, or those of the publisher, the editors and the reviewers. Any product that may be evaluated in this article, or claim that may be made by its manufacturer, is not guaranteed or endorsed by the publisher.
