# Supplementary material for: Investigating Influenza Virus Polymerase Activity in Feline Cells Based on the Influenza Virus Minigenome Replication System Driven by the Feline RNA Polymerase I Promoter
Source: Front Immunol. 2022 May 26;13:827681. doi: 10.3389/fimmu.2022.827681 (PMC9185166; doi:10.3389/fimmu.2022.827681)

**Supplementary Figure 1** The strategy for cloning and sequencing the feline RNA polI promoter.

(A) The DNA fragment containing the cat RNApolI promoter was successfully acquired by each of the three independent PCRs using three primer pairs targeting -1184-+120 (PCR1), -1094-+120 (PCR2), and -1081-+38 (PCR3) of the predicted transcription initiation site of the cat RNApolI promoter, respectively. In the control experiment, water was used as PCR template. The nucleotide sequence of PCR primers (5'→3'): [PCR1]GCTGTGCGCCCTCACGTC, GGTCCGGCTCAGCGTCAC; [PCR2] TCGGCGACGTCGGCAAGG, GGTCCGGCTCAGCGTCAC; [PCR3] CAAGGGATGTGATTTGGTCGCAGTG, CCACCGCCACATCAGAACGTGTCAG.

(B) Alignment of the cloned genomic DNA sequence in this study with the contig sequence (AANG03041276) in the GenBank database. The predicted transcription initiation site was indicated by +1 and an arrow.


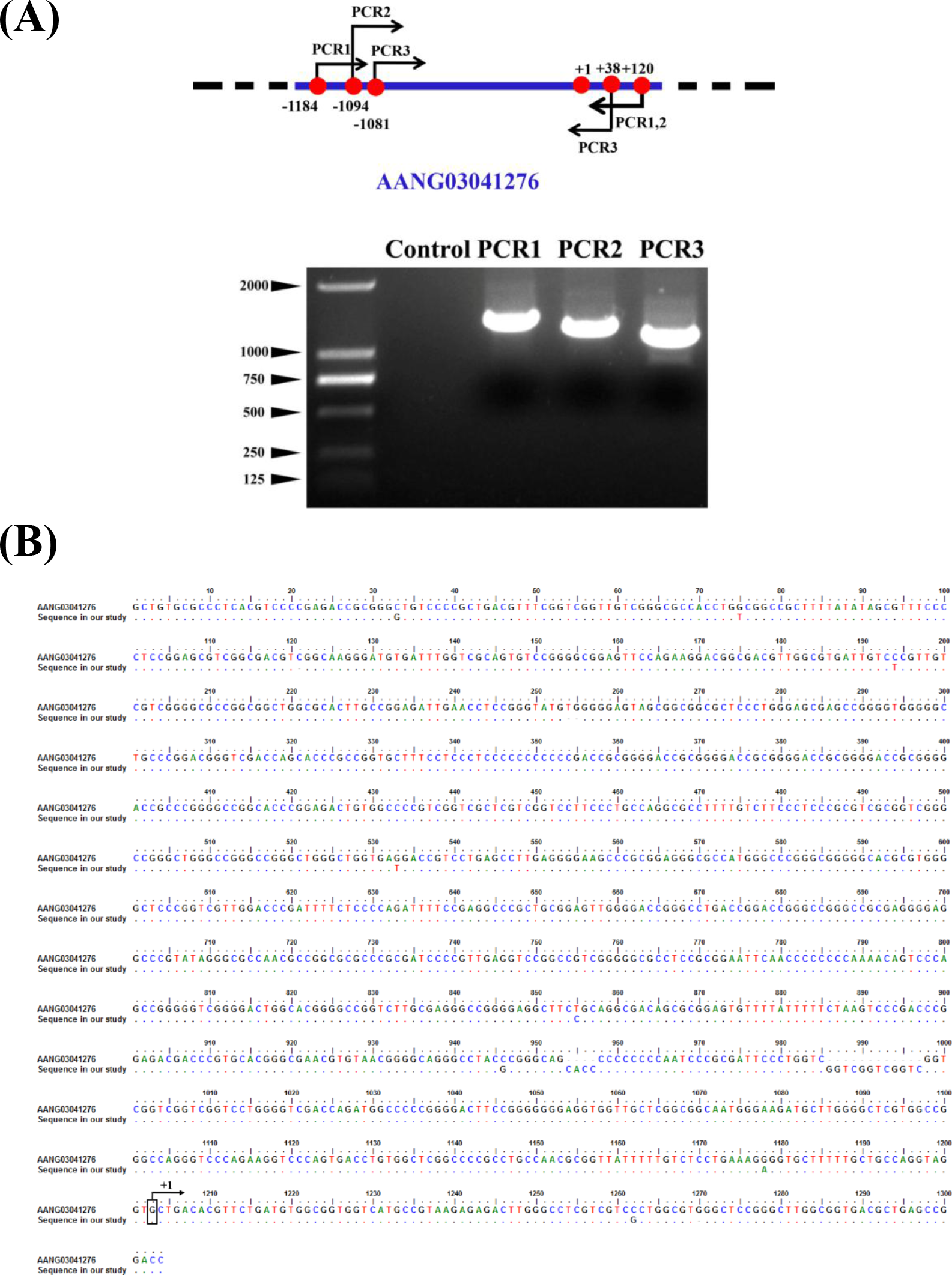

Supplement: Supplementary file 1 [file DataSheet_1.doc]
